# Predictive value of red cell distribution width for overlap syndrome in obstructive sleep apnea

**DOI:** 10.3389/fneur.2024.1415410

**Published:** 2024-05-23

**Authors:** Asli Akyol Gurses, Utku Ogan Akyildiz

**Affiliations:** ^1^Division of Clinical Neurophysiology, Department of Neurology, School of Medicine, Gazi University, Ankara, Türkiye; ^2^Neuroscience and Neurotechnology Center of Excellence (NÖROM), Gazi University, Ankara, Türkiye; ^3^Department of Neurology, School of Medicine, Adnan Menderes University, Aydin, Türkiye

**Keywords:** OSAS, chronic obstructive pulmonary disease, overlap syndrome, red cell distribution width (RDW), cardiovascular risk, inflammation

## Abstract

**Purpose:**

Obstructive sleep apnea syndrome (OSAS) and chronic obstructive pulmonary disease (COPD) are prevalent disorders, and the concurrence so-called overlap syndrome (OVS) is not rare either. Early recognition of OVS is essential because this group is more prone to cardiovascular morbidities and requires effective multidisciplinary follow-up. This study aimed to evaluate RDW in patients with severe OSAS and investigate whether it can predict OVS.

**Patients and methods:**

96 patients were retrospectively analyzed, of whom 66 were found to have severe OSAS alone and 30 OVS during diagnostic workups. Demographic, polysomnographic, and laboratory results, including RDW, were compared between groups. Multivariate logistic regression was used to determine independent associates of OVS.

**Results:**

Gender and body mass index (BMI) were similar, however, the mean age and RDW were higher in the OVS group (p:0.008, p:0.002). The increase in RDW remained significant after adjustment for age, BMI, and cardiovascular risk factors. An RDW value of >13.65% was shown to have a 78.3% sensitivity and 60% specificity for predicting OVS in severe OSAS (p:0.004).

**Conclusion:**

The results suggest that RDW can be a reliable indicator for diagnosing OVS in OSAS. It can help in identifying the subset of patients who would benefit from proper consultations and multidisciplinary follow-up, leading to appropriate treatment of each disease component and effective monitoring to prevent adverse cardiovascular outcomes.

## 1 Introduction

Obstructive sleep apnea syndrome (OSAS) and chronic obstructive pulmonary disease (COPD) are prevalent disorders among the public that cause significant cardiovascular morbidity and, impairment in patients’ quality of life (QOL). Given the prevalence rates of 2–7% for OSAS and 10% for COPD among adults; they also give rise to a significant burden on health services (1, 2). OSAS generally presents with the following symptomatological triad of snoring, excessive daytime sleepiness, and witnessed apnea due to complete or partial repetitive restrictions of the upper airway during sleep, which results in a typical oxygenation failure so-called “cyclic intermittent hypoxia. COPD is characterized by progressive airflow limitation throughout the pulmonary airways due to an abnormal inflammatory response to prolonged exposure to noxious particles and gases, of which tobacco and its products constitute the major proportion, and it is accompanied by continuous baseline hypoxia (3). The co-existence of both diseases so-called “overlap syndrome (OVS)” is reported up to the rates of 0.5–1% over 40 years of age (4), and such frequency of concurrence makes the possibility of coincidence unlikely because underlying mechanisms regarding the two are known to interact in several ways. For instance, patients with COPD are more likely to develop OSAS because of re-existing inflammation throughout the upper airway due to smoking; and among smokers, OSAS facilitates a similar process that leads to COPD because of lung damage arising from chronic intermittent hypoxia and oxidative stress (5).

Regardless of how the cause-effect relationship works, recognizing OVS as soon as possible in a patient being evaluated for sleep-related breathing disorder is essential; since this subset of patients is subjected to a greater magnitude of hypoxic exposure and systemic inflammation. They are more prone to cardiovascular consequences, facing more comorbidities and all-cause mortality (6–8), and may benefit from immediate appropriate management.

Red blood cell distribution width (RDW) is a simple and easily accessible marker from routine hemogram analysis and has been demonstrated to reflect the inflammatory state, as well as cardiovascular risk and prognosis in numerous disorders, including stroke, atrial fibrillation, and acute myocardial infarction (9–11). A number of studies also denoted its association with the severity of either OSAS or COPD (12–15). However, no studies yet evaluated the predictive value of RDW for OVS.

In this retrospective study, we aimed to evaluate RDW in patients with severe OSAS and to investigate whether it can predict OVS.

## 2 Materials and methods

### 2.1 Patients

Medical records of patients diagnosed with severe OSAS after full-night diagnostic polysomnography between June 2017 and February 2019 at a single center were retrospectively reviewed. Age, body mass index (BMI), comorbidities including hypertension, diabetes mellitus and dyslipidemia (which were accounted as cardiovascular risk factors -CVR-), scores of Epworth Sleepiness Scale (ESS), number of nocturia episodes, smoking history, red cell indices obtained from hemogram studies [hemoglobin (Hb), hematocrit (Hct), red blood cell count (RBC), mean corpuscular volume (MCV), and red cell distribution width (RDW)], and results of diagnostic polysomnography [sleep parameters including total sleep time (TST), sleep efficiency (SE), proportion of N3 and rapid eye movement (REM) stage (N3% and REM%), arousal index (AI); and respiratory parameters including apnea-hypopnea index (AHI), number of apneas, oxygen desaturation index (ODI), desaturation duration (spO2 < 90%), average spO2, minimum spO2, and nocturnal heart rate (NHR)] were recorded. Patients with a history of overt cardiac and cerebrovascular diseases [acute myocardial infarction (MI), arrhythmias and atrial fibrillation (AF), congestive heart failure (HF), and stroke] (16), pulmonary diseases other than COPD (asthma, cystic fibrosis, interstitial lung disease, and etc.), hematological and autoimmune systemic disorders; active infectious conditions during blood sampling, medications that could potentially affect hemogram parameters and the ones with obesity hypoventilation syndrome were excluded. While smoking history is a prerequisite for COPD, patients with a negative history of smoking were also excluded. The routine workup procedure in our clinical neurophysiology and sleep disorders unit for patients with complaints suggestive of OSAS included both chest and ENT (ear-nose-throat) consultations to disclose any accompanying structural and pulmonary pathologies that could mimic or exaggerate the symptoms of OSAS. Taken together, the results of diagnostic polysomnography and relative consultations, including clinical assessment and pulmonary function tests applied in accordance with the Global Initiative for Obstructive Lung Disease (GOLD) criteria, classified patients as having severe OSAS alone or severe OSAS together with COPD (overlap syndrome [OVS]) (17). Since red cell parameters are widely influenced by the magnitude of hypoxia, patients who were found to have a severe grade of OSAS after PSG (AHI ≥ 30/h) were given the priority during this study, to compare groups as similar as possible in terms of hypoxic exposure. The study protocol was approved by the local ethics committee and since the design was characterized by retrospective chart review, the need for informed consent was waived.

### 2.2 Laboratory parameters

Blood samples for routine hemogram studies were collected in EDTA tubes during the first outpatient clinic visit. The samples were automatically analyzed using the SYSMEX XE-2100 automated hematology analyzer (Sysmex Corporation, Kobe, Kansai, Japan). Red cell indices including Hb, Hct, RBC, MCV, and RDW were recorded. The normal reference range of our laboratory was as follows: Hb: 13.5–17 mg/dL for males and 12–15 mg/dL for females; Hct: 40–50% for males and 35–45% for females; RBC: 4.5–5.5×106/μL for males and 3.5–5.5×106/μL for females; MCV: 80–100 fL (for both sexes) and RDW: 12–14.5% (for both sexes).

### 2.3 Polysomnography

Overnight polysomnography was performed in all patients using the Nox Medical Program, which includes a six-channel electroencephalogram (EEG), electrooculogram (EOG), electrocardiogram (ECG), chin and leg electromyogram (EMG), oronasal thermal and nasal airflow, thoracic and abdominal respiratory effort, body position, and pulse oximetry. Data were scored manually by the same sleep specialist and clinical neurophysiologist (Dr. AAG) according to the AASM 2014 v2.4 guidelines (18). A respiratory event was scored as apnea in case of ≥90% cessation of airflow observed on the thermistor, and as hypopnea when a ≥ 30% reduction in airflow was detected on nasal airflow together with ≥3% desaturation on oximetry or arousal on EEG, both of which lasted at least 10 s. Patients with an apnea-hypopnea index of ≥30 were classified as having severe OSAS (19).

### 2.4 Statistical analysis

Statistical analyses were conducted using IBM SPSS version 20 (SPSS, Armonk, NY, United States). The Shapiro–Wilk test was utilized to assess the normality of the numeric data. Continuous variables were presented as mean ± SD or median (minimum-maximum) based on normality. Categorical variables were expressed as percentages and analyzed using the chi-square test. Pairwise comparisons were performed using an independent sample t-test or Mann–Whitney U test. Multivariate logistic regression was employed to identify independent associations with OVS, and an ROC curve analysis was used to determine the predictive potential of RDW for OVS. A two-tailed *p*-value of less than 0.05 was considered statistically significant.

## 3 Results

Between June 2017 and February 2019, 212 patients were diagnosed with severe OSAS following full-night diagnostic polysomnography in our Sleep Disorders Unit. Out of these patients, 98 had no history of smoking, while 16 out of the remaining 114 had a positive history for at least one of the mentioned cardio-cerebrovascular diseases (see “2.1 patients” section), and 2 had obesity-hypoventilation syndrome. A total of 96 patients who met the inclusion criteria were analyzed.

Out of the total study population, 87 patients were male (91%) and nine were female (7%). The mean age was 50.25 ± 13.39 years, BMI was 35.17 ± 7.06 kg/m^2^ and AHI was 66.45 ± 18.69 /hour. The median score of ESS was 6 (range: 0–17) and number of nocturia episodes was two (range: 0–8). In terms of cardiovascular risk factors (CVR), the most prevalent comorbidities were hypertension (38%), diabetes mellitus (26%), and dyslipidemia (21%). None of the participants had a history of overt cardio-cerebrovascular diseases.

30 patients (31%) were found to have co-existing COPD (OVS) after pulmonary consultation, while 66 had severe OSAS alone (69%). Among 66 patients with OSAS alone, 10 cases had position-dependent OSAS, and 2 out of these 10 patients demonstrated REM-predominancy (AHI_REM_/AHI_NREM_ ≥ 2) at the same time. No cases in the OVS group had position-dependent or REM-predominant OSAS. The mean age among OVS patients was older than that of OSAS patients (52.1 ± 8.23 vs. 47.7 ± 13.68 years, p, 0.008). However, the groups were similar in terms of sex, BMI, ESS, and number of nocturia episodes. The prevalence of cardiovascular risk factors was also similar between the two groups. The baseline demographic and clinical characteristics of the two groups (OSAS vs. OVS) are summarized in Table 1.

**Table 1:** Demographic and clinical characteristics of the patients with OSAS alone and overlap syndrome.

Variable	OSAS alone (n:66)	Overlap synd. (n:30)	*p* value
Age (yrs.)	47.70 ± 13.68	52.10 ± 8.23	0.008*
Male gender (*n*, %)	60 (91%)	27 (90%)	0.887
BMI (kg/m^2^)	34.60 ± 6.95	37.84 ± 8.52	0.073
Nocturia	2 (0–8)	2 (0–8)	0.239
ESS	6 (0–16)	7 (3–17)	0.149
Hypertension (*n*, %)	24 (36%)	12 (40%)	0.733
DM (*n*, %)	16 (24%)	9 (30%)	0.551
Dyslipidemia (*n*, %)	13 (20%)	7 (23%)	0.684

OSAS, obstructive sleep apnea syndrome; BMI, body-mass index; ESS, Eppworth Sleepiness Scale; DM, diabetes mellitus.*p < 0.05 denotes statistical significance.

The polysomnographic analysis showed that patients with OVS had decreased total sleep duration and sleep efficiency (p:0.012 and p:0.003, respectively), as well as a decreased proportion of N3 and REM sleep (*p* < 0.001 and p:0.041, respectively). Additionally, patients with OVS had a higher arousal index than patients with OSAS alone (*p* < 0.004). In terms of respiratory parameters, the oxygen desaturation index (ODI) and desaturation duration (time spent <90% SpO2) were significantly increased in the OVS group (p:0.036 and p:0.029, respectively). In contrast, AHI, total apnea count, levels of minimum and average SpO2, and nocturnal heart rate were similar between the two (Table 2).

**Table 2:** Polysomnographic and hemogram analyses of the patients with OSAS alone and overlap syndrome.

Variable	OSAS alone (n:66)	Overlap synd. (n:30)	*p* value
Polysomnography / sleep characteristics			
TST (min.)	350.5 (125.4–465.5)	291 (117.5–439.5)	0.012*
SE (%)	75.6 (29.9–95.5)	66.3 (26.8–85)	0.003*
N3 (%)	7.5 (0–38.3)	0.5 (0–22.6)	<0.001*
REM (%)	6.9 (0–38)	4.9 (0–18.3)	0.041*
Arousal index	39.4 (5.3–113.9)	59 (23.2–90.5)	0.004*
Polysomnography / respiratory parameters			
AHI	65.68 ± 19.10	69.43 ± 17.51	0.341
N_apnea_	178 (1–619)	91 (1–712)	0.639
ODI	73.03 ± 21.00	82.69 ± 22.84	0.036*
Desat. duration	21.1 (0–71.3)	32.31 (1–100)	0.029*
Average spO2 (%)	91.4 (79.5–96)	90.8 (71.8–94.5)	0.150
Min. spO2 (%)	72.39 ± 10.51	72.38 ± 9.17	0.796
Nocturnal HR	68.8 (51.2–105.5)	70.5 (54.5–108.5)	0.233
Hemogram / red cell indices			
Hb (mg/dL)	14.99 ± 1.02	15.13 ± 1.09	0.599
Hct (%)	44.12 ± 2.53	44.80 ± 3.02	0.239
RBC (10^6^/μL)	5.18 ± 0.37	5.24 ± 0.39	0.573
MCV (fL)	85.31 ± 4.32	85.94 ± 3.85	0.554
RDW (%)	13.55 ± 0.76	14.19 ± 0.88	0.002*

TST, total sleep time; min., minutes; SE, sleep efficiency; REM, rapid eye movement; AHI, apnea-hypopnea index; N_apnea_, total number of apneas; ODI, oxygen desaturation index; Desat. duration, desaturation duration (Time spent < 90% spO2); Min. spO2, minimum spO2; Nocturnal HR, nocturnal heart rate; Hb, hemoglobin (normal range: 12–17 mg/dL); RBC, red blood cell (normal range: 3.5–5.5 10^6^/μL); MCV, mean corpuscular volume (80–100 fL); RDW, red cell distribution width (12–14.5%).*p < 0.05 denotes statistical significance.

Red cell indices obtained from hemogram studies demonstrated no difference in Hb, Hct, RBC count, and MCV values between the patients with OSAS alone and OVS. However, RDW was significantly higher in the OVS group, when compared to OSAS alone (14.19 ± 0.88% vs. 13.55 ± 0.76%, p:0.002) (Table 2). Univariate analysis demonstrated the significance of both age and RDW in the presence of an OVS. Nevertheless, multivariate regression analysis, including age, BMI, CVR, and RDW, revealed that RDW was the only significant coefficient associated with OVS in severe OSAS (p:0.035) (Table 3).

**Table 3:** Coefficients regarding independent associates of overlap syndrome.

Variables	Univariate analysis			Multivariate analysis		
	Exp (B)	95% CI	p value	Exp (B)	95% CI	*p* value
Age	1.047	1.011–1.085	0.010*	1.040	0.987–1.097	0.140
BMI	1.057	0.993–1.126	0.083	1.049	0.968–1.138	0.243
CVR	1.800	0.749–4.325	0.189	1.143	0.333–3.926	0.832
RDW	2.553	1.329–4.905	0.005*	2.097	1.054–4.172	0.035*

BMI, body-mass index; CVR, cardiovascular risk factor (presence of at least one of the following; hypertension, diabetes mellitus, and dyslipidemia); RDW, red cell distribution width. *p < 0.05 denotes statistical significance.

**Figure 1:**
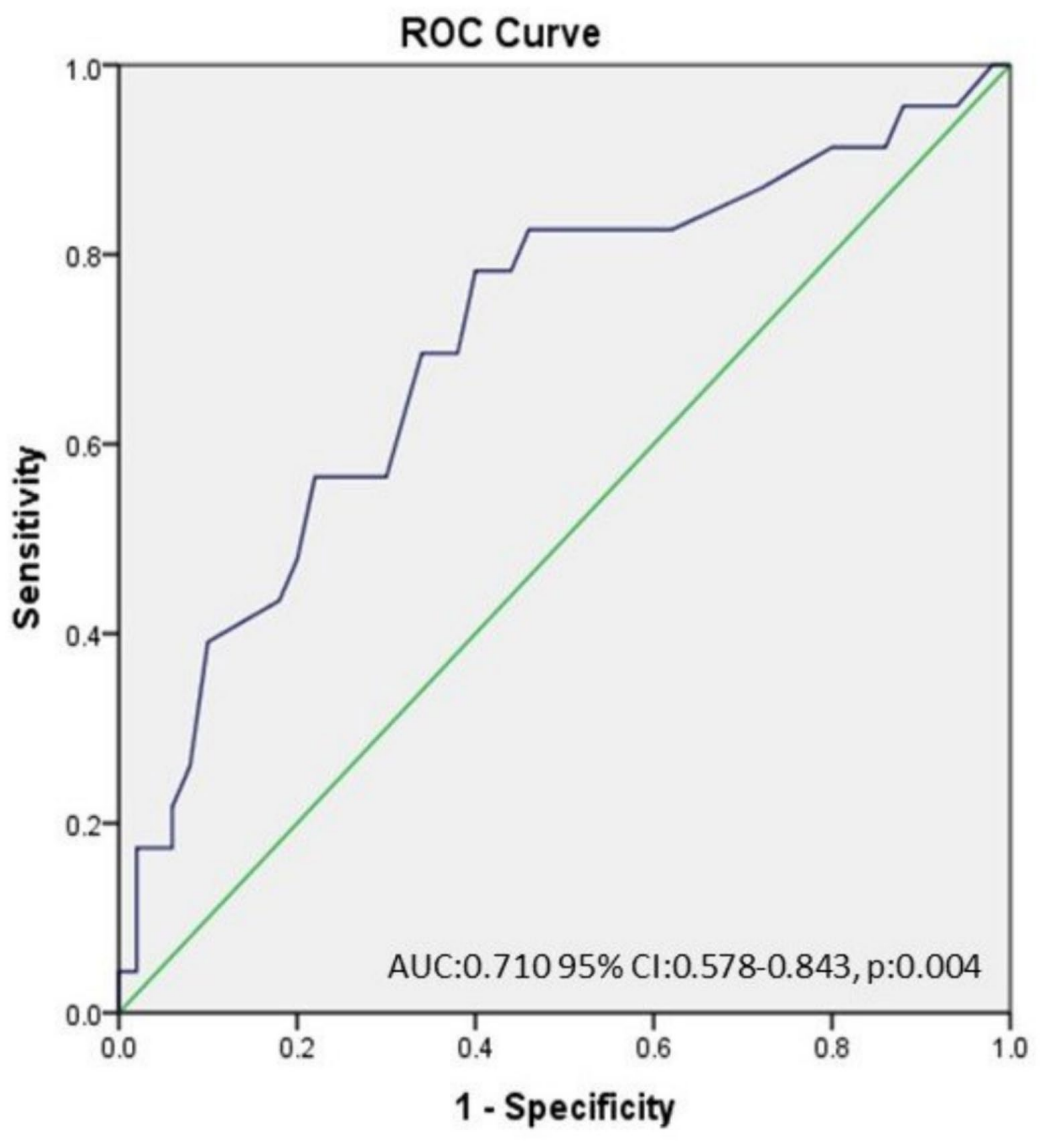
The ROC curve analysis demonstrated that an RDW value of >13.65 has 78.3% sensitivity and 60% specificity for the prediction of OVS among severe OSAS patients. (AUC: 0.710 95% CI: 0.578–0.843, p:0.004).

The ROC curve analysis demonstrated that an RDW value of >13.65 has 78.3% sensitivity and 60% specificity for the prediction of OVS among severe OSA patients (AUC: 0.710 95% CI: 0.578–0.843, p:0.004) (Figure 1).

## 4 Discussion

Our study showed that patients with OVS had elevated levels of RDW compared to those with severe OSAS alone. This association remained significant even after adjusting for potential confounders. An RDW value of >13.65% was found to have 78.3% sensitivity and 60% specificity for predicting OVS in severe OSA. Our results also confirmed worse sleep quality and pronounced desaturation during sleep in this population.

OSAS and COPD are both frequent diseases with marked negative impacts on patients’ QOLs, and they can occur together up to the rates of 11–23% (20). Concurrence of OSAS and COPD in the same patient is called OVS (21), and due to a couple of deteriorating mechanisms, including but not limited to systemic inflammation, endothelial dysfunction, and tonic increase in sympathetic activity (22), OVS is associated with a higher number of vascular risk factors; as well as an increased rate of cardiac/cerebrovascular diseases (23). For instance; numerous pathways, including nuclear factor kappa-B (NF-κB), tumor necrosis factor-alpha (TNF-α), C-reactive protein (CRP), and interleukin 6 (IL-6), are known to be involved in the development and maintenance of a hypoxia-induced inflammatory background in OSAS. In addition to cyclic intermittent hypoxia, which is a characteristic of OSAS, sustained hypoxemia in coexisting COPD, seems to activate other molecules, such as transcription factors produced via HIF-1 alpha-mediated pathways (5). This persistent inflammatory exposure seems to contribute to an amplified process of atherosclerosis, along with numerous cardiovascular risks and diseases in overt form, of which the latter is documented to be more pronounced in OVS (6). In line with this, Papachatzakis et al. demonstrated a higher rate of multiple comorbidities (≥4) among patients with OVS, when compared to the ones with OSAS alone (28.95% vs. 10.52%) recently (7). A few years later, Voulgaris et al. reported a higher prevalence of overt cardiovascular disease (CVD) in the OVS group than in the OSAS alone group (23.9% vs. 13.5%) (6). Of note, the researchers identified arterial hypertension, diabetes mellitus, and dyslipidemia as the most common comorbidities, except for overt CVD, which was initially excluded from our study due to the methodology. Taken together with the molecular and clinical evidence, OVS should be recognized early and the diagnosis should not be missed.

However, underdiagnosis regarding either OSAS or COPD and OVS as a result, is not rare (20). Although there have been some efforts to identify an inflammatory molecule as a distinguishing biomarker, the number of relevant studies is quite limited. In 2013, Nural et al. evaluated serum CRP, TNFα, and asymmetric dimethylarginine (ADMA) levels among three groups of patients with OSAS alone, COPD, and OVS in the pre- and post-CPAP (continuous positive airway pressure) periods; and identified lower levels of pre-CPAP ADMA in the OSAS alone group than in the COPD group (24). The authors detected no difference among the groups in terms of CRP and TNFα levels; however, CRP was found to improve after CPAP in both groups of patients with OSAS alone and OVS. One year later, comparable results from a similar study population were reported by Wang et al. with the exception that both TNF-α and CRP levels were ameliorated following CPAP treatment (25). More recently, Archontogeorgis et al. assessed platelet indices between healthy controls and patients with either OSAS or OVS and demonstrated that the mean platelet volume (MPV) and platelet distribution width (PDW) were higher in both patient groups than in controls. The MPV in patients with OVS was also higher than that with OSAS alone (26). To sum up the results, these limited data indicate a worse inflammatory status in patients with COPD, either in pure or combined form as a component of OVS, as to OSAS alone.

The findings of the current study pointed to a similar conclusion through a different marker, and the results revealed that patients with OVS had increased RDW when compared to those with OSAS alone. RDW is a simple and easily accessible marker obtained from routine hemogram analysis and indicates the heterogeneity of the RBC size. Its alterations have served as a distinguishing mark between anemia subtypes for years; however, accumulating evidence from recent literature has discovered that it reflects far beyond (9–11, 27). Its association with the incidence and severity of numerous disorders such as atherosclerosis, myocardial infarction, heart failure, hypertension, and AF has been reported in many studies (28). Similarly, the diagnostic and prognostic values in ischemic cerebrovascular disease, acute coronary syndrome, and peripheral artery disease have been underscored in many other ones (29). A number of papers also denoted its association with severity of either OSAS or COPD. For instance, Kallel et al. demonstrated a positive correlation between the apnea-hypopnea index and RDW and identified it as an independent predictor of cardiovascular disease in OSAS (12). Another cross-sectional study that included elderly patients with OSAS and controls reported higher levels of RDW in patients with severe disease than with other disease grades and controls (13). Regarding COPD; elevated RDW level was found to be associated with mortality risk in 270 stable patients (14); whereas the association was either true for prolongation of hospital stay among patients with acute exacerbations (15). Despite considerable data on each disease, no study has evaluated the predictive value of RDW in OVS, and our study represents a first in this regard.

RDW could be influenced by age, sex, and genetic factors besides hematological diseases (30). A positive history of hematological disorders, as well as overt cardiac and cerebrovascular diseases, were among the main exclusion criteria in our study. The patient groups were similar in terms of sex and prevalence of CV risk factors. Although an age difference was present between the two groups, the significance disappeared after applying a multivariate regression model for confounders.

Considering the additional findings of the current study, it was seen that the average age was older in the OVS group. This was in line with the previous epidemiological data, which indicate a remarkable relationship between increased age and COPD prevalence (17). In a recent study by Voulgaris et al., which examined the 10-year CV disease risk between individuals with OSAS alone and OVS, a higher median age was reported for OVS patients than for OSAS cases (54 vs. 51 years, *p* < 0.05) (23). Positive smoking history and excess BMI are other well-known clinical and anthropometric variables that demonstrate positive correlation with the odds of OSAS in patients with COPD (31). However, the patients were similar in terms of both features in our study, despite a tendency towards BMI increase in the OVS group that did not reach significance.

According to sleep analyses of diagnostic polysomnography, OVS patients in the current cohort had a more disrupted sleep architecture and worse sleep quality. A reduction in TST and SE, as well as decreased amounts of REM and slow-wave sleep, are common disturbances observed in the polysomnographic studies of these patients (32). This derangement could result from dyspnea, nocturnal cough, and impaired gas exchange due to COPD, which may be exaggerated in the presence of sleep apnea, already characterized by sleep fragmentation (33), and lead to anticipation of excessive daytime sleepiness symptoms in this group. However, daytime symptoms were similar for both groups in this study. It may be attributed to the fact that other scales evaluating daytime sleepiness other than ESS were not used in the assessment of the patients. Regarding the respiratory analyses of polysomnographic records, patients with OVS in our study were found to have increased ODI and desaturation duration than OSAS alone. This was again compatible with the previous reports (6, 23, 26), as superimposed obstructive events on baseline hypoxemia are associated with more profound nocturnal oxygen desaturation during sleep in patients with OVS (5, 34). On the other side, nocturnal heart rate was found similar between the two groups in the current study, although patients with OVS were demonstrated to have higher sympathetic and lower parasympathetic modulation of heart rate variability (HRV) previously (35). This similarity may be because both groups had severe levels of OSAS at baseline, and the modulation dominance in HRV may not be reflected in the nocturnal heart rate as a direct output.

The current study had some limitations. Firstly, the retrospective design may have led to missing potential cases of individuals who might develop OVS during the follow-up period, and again it does not allow monitoring of the trend of RDW levels in response to different treatment modalities over time. Secondly, the analyses were conducted on severe OSAS patients to optimize comparison conditions, which could initially seem discouraging when generalizing the findings to other disease levels. However, the parameter “RDW” is known to be significantly affected by hypoxia, and the comparison of groups with similar levels of hypoxic exposure as possible, together with adequately controlling for other confounding factors, strengthens the reliability of the results. Co-evaluation of RDW with well-known clinical parameters and biomarkers in the follow-up of OVS could validate the effectiveness and uniqueness of its role in predicting OVS. In adition to this, prospective studies that include a larger and more diverse sample size comprising patients with different demographics, comorbidities, and severity levels of OSAS, as well as a long-term follow-up to assess the outcomes and progression of OVS, could provide valuable insights into the clinical implications of RDW as a predictive marker.

## 5 Conclusion

RDW has a reasonably good predictive value for diagnosing OVS upon evaluation of a patient with OSAS. It could be used as a guide to identify the ones who need further evaluation by pulmonologists and cardiologists. This selective approach will help to determine the appropriate treatment for each disease present in the patient and enable effective multidisciplinary monitoring to prevent adverse cardiovascular outcomes.

## Data availability statement

Data regarding this study are available upon reasonable request from the corresponding author. Requests to access the datasets should be directed to AA, akyol1984@yahoo.com.

## Ethics statement

The studies involving humans were approved by Ethics Committee of Aydin Adnan Menderes University. The studies were conducted in accordance with the local legislation and institutional requirements. The human samples used in this study were acquired from hemogram analyses of the patients which were routinely ordered as a part of a clinical evaluation on the first visit to the outpatient clinic. Written informed consent for participation was not required from the participants or the participants' legal guardians/next of kin in accordance with the national legislation and institutional requirements.

## Author contributions

AA: Writing – review & editing, Writing – original draft, Supervision, Methodology, Investigation, Formal analysis, Conceptualization. UA: Writing – review & editing, Writing – original draft, Supervision, Methodology, Conceptualization.
